# Distinct molecular features of *FLNC* mutations, associated with different clinical phenotypes

**DOI:** 10.1002/cm.21922

**Published:** 2024-09-24

**Authors:** Ekaterina S. Klimenko, Anastasia K. Zaytseva, Margarita Yu. Sorokina, Kseniia I. Perepelina, Nataliia L. Rodina, Ekaterina G. Nikitina, Kseniia S. Sukhareva, Aleksandr A. Khudiakov, Tatyana L. Vershinina, Alexey S. Muravyev, Evgeny N. Mikhaylov, Tatyana M. Pervunina, Elena S. Vasichkina, Anna A. Kostareva

**Affiliations:** ^1^ Almazov National Medical Research Centre Institute of Molecular Biology and Genetics Saint‐Petersburg Russia; ^2^ Karolinska Institutet, Department of Women's and Children's Health Karolinska University Hospital Stockholm Sweden

**Keywords:** cardiomyocyte, cardiomyopathy, electrophysiology, filamin, iPSC, transcriptome

## Abstract

Filamin С is a key an actin‐binding protein of muscle cells playing a critical role in maintaining structural integrity and sarcomere organization. *FLNC* mutations contribute to various types of cardiomyopathies and myopathies through potentially different molecular mechanisms. Here, we described the impact of two clinically distinct *FLNC* variants (R1267Q associated with arrhythmogenic cardiomyopathy and V2264M associated with restrictive cardiomyopathy) on calcium homeostasis, electrophysiology, and gene expression profile of iPSC‐derived patient‐specific cardiomyocytes. We demonstrated that R1267Q *FLNC* variant leads to greater disturbances in calcium dynamics, Nav1.5 kinetics and action potentials compared to V2264M variant. These functional characteristics were accompanied by transcriptome changes in genes linked to action potential and sodium transport as well as structural cardiomyocyte genes. We suggest distinct molecular effects of two *FLNC* variants linked to different types of cardiomyopathies in terms of myofilament structure, electrophysiology, ion channel function and intracellular calcium homeostasis providing the molecular the bases for their different clinical phenotypes.

## 1 INTRODUCTION

In recent years a growing number of actin‐binding proteins have been acknowledged as a causative for inherited human diseases. In addition to well‐known disease‐causing genes such as *INF2* for kidney disorders and neuropathies, *DIAPH1* for inherited hearing loss, and *GSN* for inherited amyloidosis, many new genes were described and recognized in association with human pathologies (Brown et al., 2010; Levy et al., 1990; Lynch et al., 1997). This opened a broad research area focused on tissue‐specific actin‐binding proteins and their role in cell proliferation and differentiation, regulation of signaling cascades, interactions with extracellular matrix, and mechanotransduction. Among such proteins are filamins—actin‐binding dimeric proteins with substantial tissue‐specific expression. Three main types of human filamins in—filamin A, B, and C, encoded correspondingly by *FLNA*, *FLNB*, and *FLNC* are associated with various human disorders (Feng & Walsh, 2004; van der Flier & Sonnenberg, 2001). Thus, mutations in *FLNA* were first described in association with Periventricular heterotopia and later on with cardiac valve disorders, and platelet pathologies (Bernstein et al., 2011; Nurden et al., 2011). Pathogenic variants in FLNB gene were described in connection to skeletal malformations and bone pathologies while mutations in *FLNC* are mainly linked to cardiac and skeletal muscle disorders (Verdonschot et al., 2020; Krakow et al., 2004; Song et al., 2022). Currently, *FLNC* gene is one of the most common genetic causes of various types of cardiomyiopathies—severe inherited disorders of cardiac muscle leading to sudden cardiac death, heart failure and poor prognosis. Currently, *FLNC* mutations contribute to all types of cardiomyopathies—hypertrophic, dilated, arrhythmogenic and restrictive. All these types of genetic cardiac disorders have different clinical features and prognosis due to distinct molecular pathogenesis. Thus, *FLNC*‐associated restrictive cardiomyopathy is mainly a consequence of intracellular protein aggregation due to dominant‐negative mutations, while dilated and arrhythmogenic cardiomyopathies result from the opposite mechanism of haploinsufficiency. Uncovering the molecular pathogenesis of different types of FLNC‐associated cardiomyopathies allows to approach more personalized therapeutical options in future using either gene therapy or modulators of proteostasis.

Earlier, we described two padiatric patients with *FLNC*‐associated cardiomyipathies (Kofeynikova et al., 2023; Muravyev et al., 2022). The first one, carrying R1267Q *FLNC* variant presented with childhood‐onset arrhythmogenic cardiomyopathy. The variant R1267Q (rs768767784) was earlier reported in population with a frequency of 1:40596 and is currently suggested to be a variant of unknown significance. Therefore, a detailed characteristic of its functional effect is of clinical and scientific importance. The second patient carrying V2264M *FLNC* variant underwent heart transplantation due to restrictive cardiomyopathy. The variant V2264M (rs1420394583) was earlier reported in ClinVar databases as pathogenic, likely pathogenic, or variant of unknown significance, is predicted to be pathogenic by several prediction programs and, being also present in patient's mother with cardiomyopathy, is treated as likely pathogenic. In a current study using patient‐specific induced pluripotent stem cell approach, functional studies, and transcriptomic analysis we confirmed distinct molecular effects of these two *FLNC* variants in terms of electrophysiology, ion channel function, and intracellular calcium homeostasis. We first demonstrated the direct effect of *FLNC mutations* on potential‐dependent sodium current properties and suggested the molecular mechanisms for *FLNC*‐associated arrhythmogenesis.

## 2 MATERIALS AND METHODS

### 2.1 Generation, cultivation, and differentiation of human hiPSCs

Human induced pluripotent stem cells (hiPSC) lines carrying *FLNC* R1267Q (FAMRCi011‐A) and *FLNC* V2264M (FAMRCi009‐A) variants were generated from patient's PBMCs using CytoTune‐iPS 2.0 Sendai Reprogramming Kit (Thermo Fisher Scientific) as previously described (Khudiakov et al., 2020). Detailed information about cell lines and characterization can be found in hiPSCreg database (https://hpscreg.eu/cell-line/FAMRCi011-A; https://hpscreg.eu/cell-line/FAMRCi009-A). A control Donor cell line AD3 was generated from HNFs purchased from Lonza (Slough, UK) using the lentiviral, non‐integrating Sendai reprogramming kit CytoTune‐iPS 2.0 Sendai Reprogramming kit (Invitrogen) according to the manufacturer's instructions (Neganova et al., 2016).

All iPSC lines were cultured on plates coated with Geltrex in Essential 8 medium (Thermo Fisher Scientific), passaged upon reaching 70%–80% confluence using ReLeSR (Stem Cell Technologies), and switched to culture media supplemented with Rock kinase inhibitor Y‐27632 (5 μM, Tocris). hiPSC differentiation in CMCs (hiPSC‐CMC) was performed by modulating Wnt pathway, applying small molecules (Lian et al., 2012) with modifications (see Supplemental Material). Metabolic selection was performed from days 12–15 of as described (Tohyama et al., 2013). For electrophysiological analysis of Nav1.5, action potential and calcium transient cells were cultured in RPMI/B27 medium until seeding on Geltrex‐coated coverslips using Tryple Select reagent (Thermo Fisher Scientific) for dissociation.

### 2.2 Immunofluorescent staining and analysis

Cells were fixed for 10 min with 4% paraformaldehyde in PBS and permeabilizing for 5 min with 0.1% Triton‐X at room temperature. The antigen retrieval method was applied for filamin C staining using the urea‐containing buffer (0.1 M Tris–HCl, 5% urea, pH 9.5) at 80°C during 5 min. The primary antibodies were applying during 1 h: anti‐cardiac alpha‐actin at a dilution of 1:100 (M874, DAKO, USA), anti‐troponin I at a dilution of 1:100 (sc‐15,368, Santa Cruz Biotechnology, Inc, USA), anti‐tropomyosin at a dilution of 1:100 (sc‐73,225, Santa Cruz Biotechnology, Inc, USA), anti‐sarcomeric alpha‐actinin at a dilution of 1:100 (A7811, Sigma‐Aldrich, USA), anti‐filamin C at a dilution of 1:50 (HPA006135, Sigma‐Aldrich, USA). The secondary antibodies were applying for 40 min at a dilution of 1:1500. The working dilution for phallodin‐488 staining was 1:1000 applied during 30 min. Images were acquired using a Zeiss Observer.Z1 microscope with ZEN 2.6 Blue edition software (Zeiss, Germany) under the same condition of shooting. Z‐disk width was measured by the intensity profile (ZEN 2.6 Blue edition) of the phalloidin staining and the alpha‐actinin staining. The width of troponin I and tropomyosin staining were measured also using profile intensity (Supplemental Figure 1). The distance between the fluorescence maximum of phalloidin staining and the nearest fluorescence minimum of cardiac actin staining was used to measure actin shift. The fluorescent intensity of filamin C was normalized to the alpha‐actinin fluorescent intensity.

### 2.3 Western blot

Total protein fraction was extracted from cells seeded on cultural plate by resuspending cells in RIPA buffer with protease inhibitor cocktail (Roche, USA). Cells lysates were incubated for 30 min on ice, following 16,000 g centrifugation to separate protein fraction. Before loading protein lysate into the acrylamide gel, samples were incubated in 2× Laemmli buffer with β‐mercaptoethanol at 95°С for 5 min. To separate proteins by molecular weight we used 10% acrylamide gel. SDS‐PAGE electrophoresis was performed at 20 and 40 mA for stacking and resolving gels, respectively. Protein transfer was performed using nitrocellulose membrane with 0.45 μm pore size (Aplichem, USA). After protein transfer membrane were blocked in 5% milk solution followed by overnight incubation at +4°C in primary anti‐STIM1 (SC‐166840, Santa Cruz, USA) and anti‐TRPC1 (SC‐133076, Santa Cruz, USA) antibody diluted 1:1000. Secondary anti‐mouse HRP antibody (BioRad, USA) was used at concentration 1:20,000. The obtained data was processed using the quantification analysis module of Fusion software. The evaluation of the amount of protein in the band was performed by estimating the optical density of pixels in the selected area of the sample. Protein level for each sample was evaluated relative to control sample and to Ponceau staining for protein loading. Control sample was assigned to 1.0.

### 2.4 Calcium dynamic with stimulation

Registration of calcium transients was carried out by electrical stimulation on IonOptix setup (Westwood, MA) using calcium‐sensitive fluorescent dye Fura‐2 AM (Thermo Fisher Scientific, USA). Prior to imaging cells were incubated in 2 μM Fura‐2 AM in calcium‐containing buffer for 30 min at 37°C. Cells were paced at 0.2 Hz with a 10 V square pulse using a MyoPacer field stimulator (IonOptix, Westwood, MA). Transients from one cell were recorded for 2–3 min and averaged. For each cells the following parameters were recorded: transient amplitude, transient duration (same with time to 90% baseline), pre‐peak and post‐peak parameters groups: time to 10%, 50%, 100% peak (Tp10%, Tp50%, Tp) and release velocity; time to 10%, 50% baseline (Tb10%, Tb50%), time to baseline (transient duration–Tp) and return velocity (Supplemental Figure 2).

### 2.5 Electrophysiology and patch‐clamp data analyses

Sodium currents and action potentials were recorded using whole‐cell patch‐clamp configuration at room temperature (Khudiakov et al., 2020). Microelectrodes were manufactured using a puller (P‐1000, Sutter Instrument) with resistance varied from 2.0 to 3.5 MΩ. Data acquisition and junction potential correction were done using Axopatch 200B amplifier and Clampfit software version 10.3 (Molecular Devices Corporation). Currents were acquired at 20–50 kHz and low‐pass filtered at 5 kHz using an analog‐to‐digital interface (Digidata 1440A acquisition system, Molecular Devices Corporation). The series resistance was compensated at 75%–80%. All pulse protocols were applied more than 5 min after membrane rupture. APs were elicited in current‐clamp mode at 1 Hz frequency by 3 ms, 1.2× threshold current pulses through the patch pipette.

Current densities at each test potential were obtained by dividing the *I*_Na_ by cell capacitance. Cell capacitance values did not differ significantly between experimental groups. Pulse protocols were applied with a holding potential of −100 mV. Current–voltage (*I*–*V*) curves were assessed by depolarizing voltage steps from −80 to 60 mV during 40 ms in 5 mV increments at 1 Hz frequency. The maximal *I*_Na_ at each voltage was obtained and the corresponding conductance (*G*) was calculated using equation *G* = *I*_Na_/(*V* − *V*_rev_), where *V* is the voltage test. The normalized *G* values were plotted against the voltage, and the *G*–*V* curves, which characterize the steady‐state activation, were fitted to the Boltzmann function *G*/*G*_max_ = 1/(1 + exp ((*V*1/2 − *V*)/*k*)), where *G*_max_ is the maximal sodium conductance, *V*1/2 is the potential of half‐maximal activation, and k is the slope factor. The voltage dependence of the steady‐state inactivation was tested by measuring *I*_Na_ elicited by a 20 ms step to −15 mV after a prepulse of 500 ms ranging from −120 to 0 mV in 5 mV steps. The normalized *I*_Na_ was plotted against the prepulse voltage. The steady‐state inactivation curves were fitted with the Boltzmann function. The AP amplitude (APA), and AP duration (APD) at 30%, 50%, and 90% repolarization (APD30, APD50, and APD90, respectively) were analyzed using IgorPro8 software (WaveMetrics).

### 2.6 RNA sequencing and bioinformatics analysis

Total RNA was extracted with ExtractRNA reagent (Evrogen) according to manufacturer's instructions and quantified using Qubit 2.0 fluorometer (Life Technologies). One microgram (1 μg) of RNA was used for library synthesis. The RNA libraries were generated using the TruSeq Stranded mRNA kit (Illumina, USA), following the manufacturer's protocol. Library quantification was conducted using the 4150 TapeStation system (Agilent) with High Sensitivity DNA ScreenTape. Sequencing was performed on the Illumina NextSeq 2000 platform with 100 cycles. Quality assessment of the raw reads was performed using FastQC (v0.11.9), and the fastp program (v0.12.4) was utilized to eliminate overrepresented polyG sequences. Then, reads were aligned to the human genome using the STAR aligner (v2.7.9) with the GRCh38.p13 reference genome and GENCODE annotation (gencode.v41.primary assembly). Mapped reads were counted using the featureCounts program (v2.0.3). Differential gene expression analysis was carried out with the RStudio package DESeq2 (v1.40.1). Gene *p*‐values were adjusted using the Benjamini–Hochberg procedure and filtered with *p*.adj <0.05; only genes with a |log2 fold change| >1 were considered differentially expressed. Subsequently, Gene Set Enrichment Analysis and GO Enrichment Analysis of gene sets were performed to identify signaling pathways. Visualization of gene expression levels of DEGs was performed using the Phantasus tool (v1.21.5). The raw RNA sequence data and counts table are openly available in GEO database (GEO: GSE260653 https://www.ncbi.nlm.nih.gov/geo/query/acc.cgi?acc=GSE260653).

### 2.7 Statistics

At least three independent experiments and at least three independent cardiac differentiations were performed for each measurement. Statistical analysis was performed using GraphPad Prism version 5.00 for Windows (GraphPad Software, www.graphpad.com). For comparison of two groups the Mann–Whitney test was used. An alpha level of 0.05 was used for all statistical analyses. Data are presented as mean ± SEM.

## 3 RESULTS

### 3.1 General transcriptomic analysis

**FIGURE 1:**
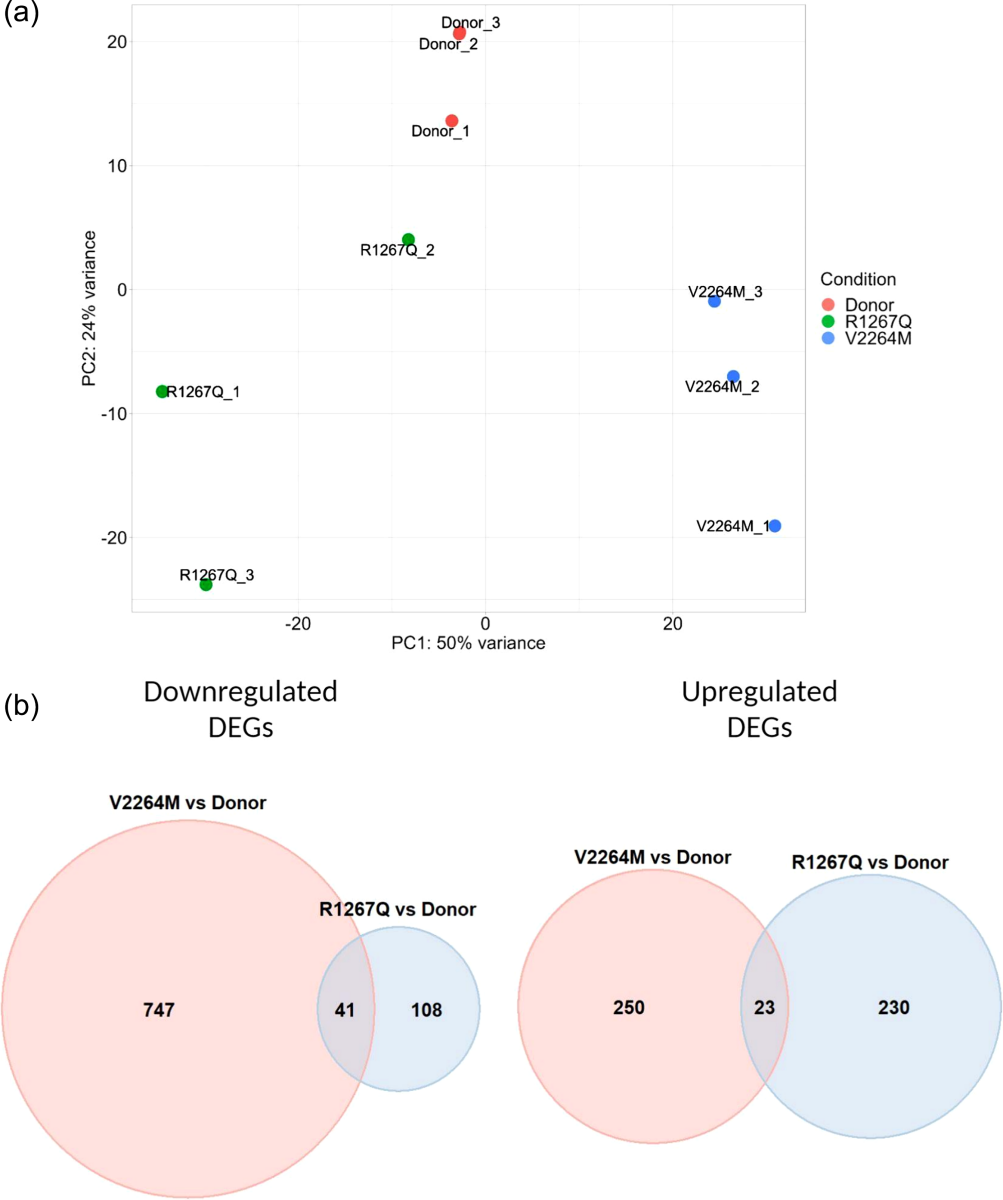
Summary of transcriptome analysis results. (a) Results of a principal component analysis (PCA) of transformed count data from RNA‐seq using DESeq2 (varianceStabilizingtransformation). Samples are colored according to FLNC mutation status. (b) Venn diagram of downregulated and upregulated differentially expressed genes of two sample comparisons: R1267Q hiPSC‐CMC versus Donor hiPSC‐CMC and V2264M hiPSC‐CMC versus Donor hiPSC‐CMC.

Principal component analysis of transformed RNA‐Seq count data obtained from R1267Q hiPSC‐CMC, V2264M hiPSC‐CMC, and Donor hiPSC‐CMC demonstrated clear differences between sample groups (Figure 1a). Differential expression analysis was performed between pairs of samples: R1267Q versus Donor and V2264M versus Donor. The analysis used the following criteria for selecting differentially expressed genes (DEGs): |LFC| >1 and *p*.adj <0.05. Venn diagram of downregulated and upregulated differentially expressed genes detected in the pairwise comparisons is shown in Figure 1b. For each mutation, we identified 41 shared downregulated DEGs and 23 shared upregulated DEGs (see Supplemental Table 1). Accordingly, further transcriptomic and functional analysis was performed separately for each mutation to uncover the molecular mechanisms typical for each *FLNC* variant.

### 3.2 Characterization of R1267Q *FLNC* variant

#### 3.2.1 Calcium imaging

**FIGURE 2:**
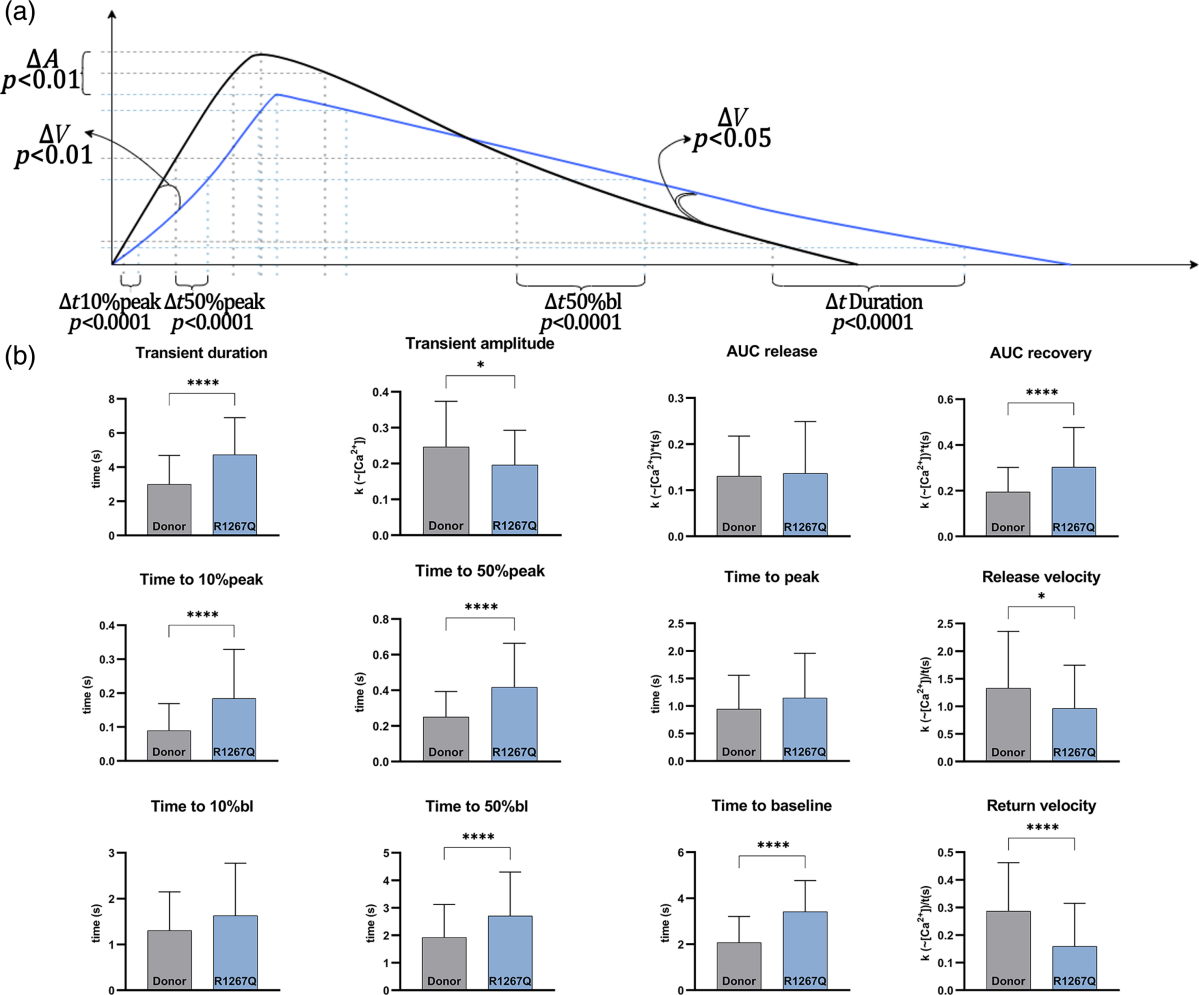
Calcium transients of Donor hiPSC‐CMC and R1267Q hiPSC‐CMC. Black plot—health donor hiPSC‐CMC (*n* = 129); blue plot—R1267Q hiPSC‐CMC (*n* = 78). (a) Dynamics of calcium transients. Dotted lines—time points (10%, 50%, 90%, and peak). *A*—calcium transient amplitude; *V*—velocity. Different properties are specified; *p*‐value marked under each parameter. (b) Parameters of calcium transient. AUC—area under curve. *p*‐value: * −<0.05; ** −<0.01; *** −<0.001; **** − <0.0001. The columns are presented as the mean and the bars correspond to SD.

For R1267Q hiPSC‐CMC most parameters of calcium transients were significantly changed (Figure 2a). Transient duration was increased along with time of baseline recovery (Figure 2b). Tp10% and Tp50% were significantly higher, but the difference has been leveled out at peak reach time point. Calcium return velocity was significantly decreased along with increase in transient duration. Moreover, we detected a significant decrease in release velocity that may be related to decreased transient amplitude under the same peak reach duration compared to Donor hiPSC‐CMC. Due to the prolongation of all duration parameters and low return velocity, an increase of AUC recovery was observed.

Thus, R1267Q hiPSC‐CMC revealed increased calcium transient duration along with decreased transient amplitude, release, and return velocity. In addition, we performed Western blot analysis using antibodies against storage‐operated calcium entry‐linked proteins STIM1 and TRPC1 and detected the tendency although not significant for decrease in their amount (Supplemental Figure 3). This suggests that alterations of calcium homeostasis in R1267Q hiPSC‐CMC might be present both on functional and protein levels.

#### 3.2.2 Electrophysiology

**FIGURE 3:**
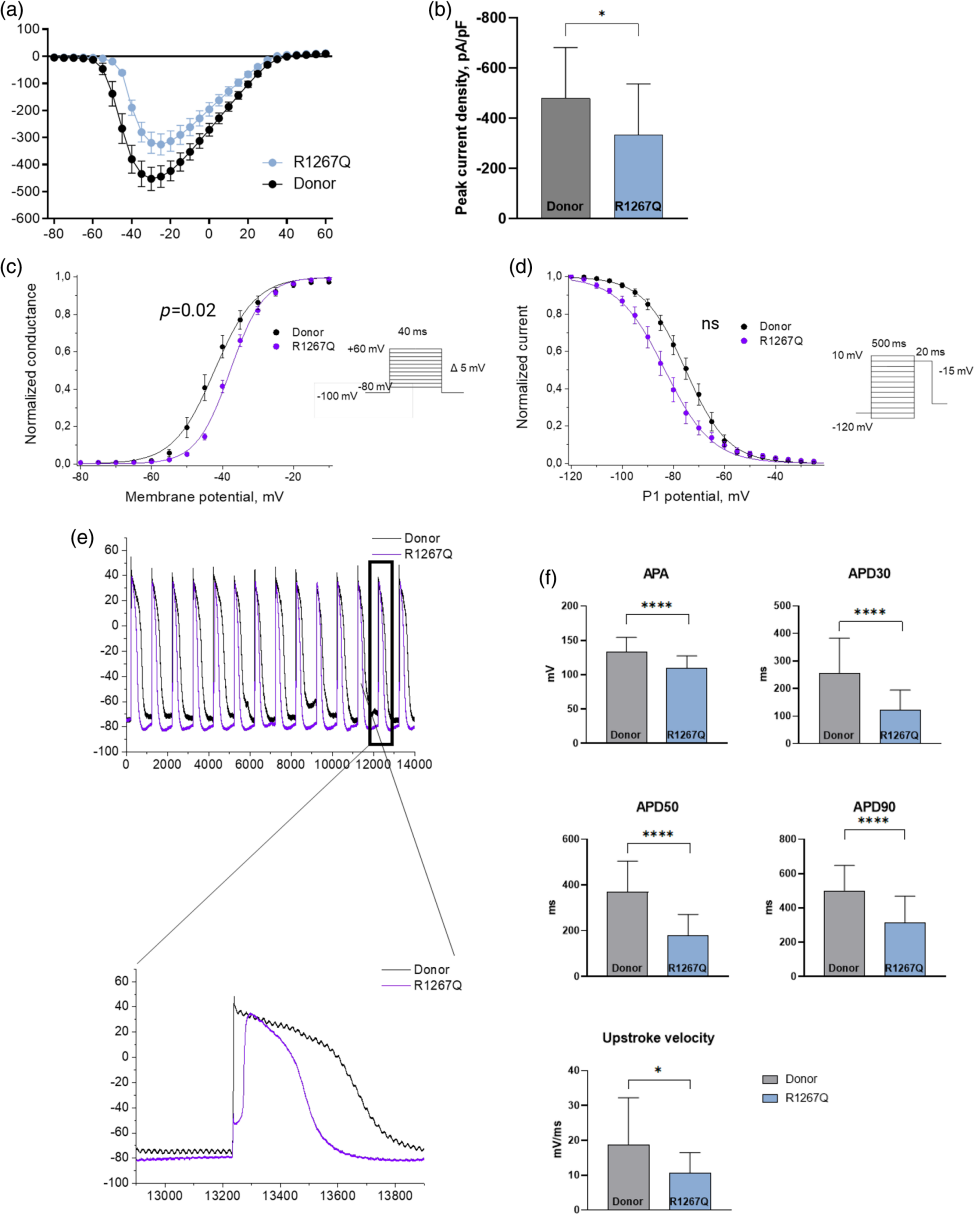
Biophysical characteristics of sodium current and action potential in R1267Q hiPSC‐CMC. Black plot—health donor hiPSC‐CMC (*n* = 23); blue plot—R1267Q hiPSC‐CMC (*n* = 36). (a) Volt–Ampere characteristics of sodium current. (b) Peak of sodium current density. (c) Steady‐state activation of sodium current. (d) Steady‐state inactivation of sodium current. (e) Representative traces of action potentials. (f) Parameters of action potential. *p*‐value: * −<0.05; ** −<0.01; *** −<0.001; **** −<0.0001. The columns are presented as the mean and the bars correspond to SD.

Typical sodium current was registered in all groups of cells (Supplemental Figure 4). We detected a significant decrease in peak sodium current in R1267Q hiPSC‐CMC compared to Donor hiPSC‐CMC (Figure 3a, b). As a next step, we examined the steady‐state activation and inactivation of sodium current. FLNC‐R1267Q variant lead to positive 5 mV shift of steady‐state activation, indicating that observed sodium current density decrease could be explained by impaired activation (Figure 3c). At the same time, it does not result in any significant changes in inactivation characteristics compared to donor hiPSC‐CMC (Figure 3d). In addition, we detected reduced action potential amplitude, action potential duration (APD30, APD50, and APD90) and upstroke velocity, as well as increased depolarization time in R1267Q hiPSC‐CMC (Figure 3e, f) compared to Donor hiPSC‐CMC. These changes are in consistent with impaired activation of sodium channels in R1267Q hiPSC‐CMC.

#### 3.2.3 Pathway analysis of transcriptional changes in R1267Q hiPSC‐CMC

**FIGURE 4:**
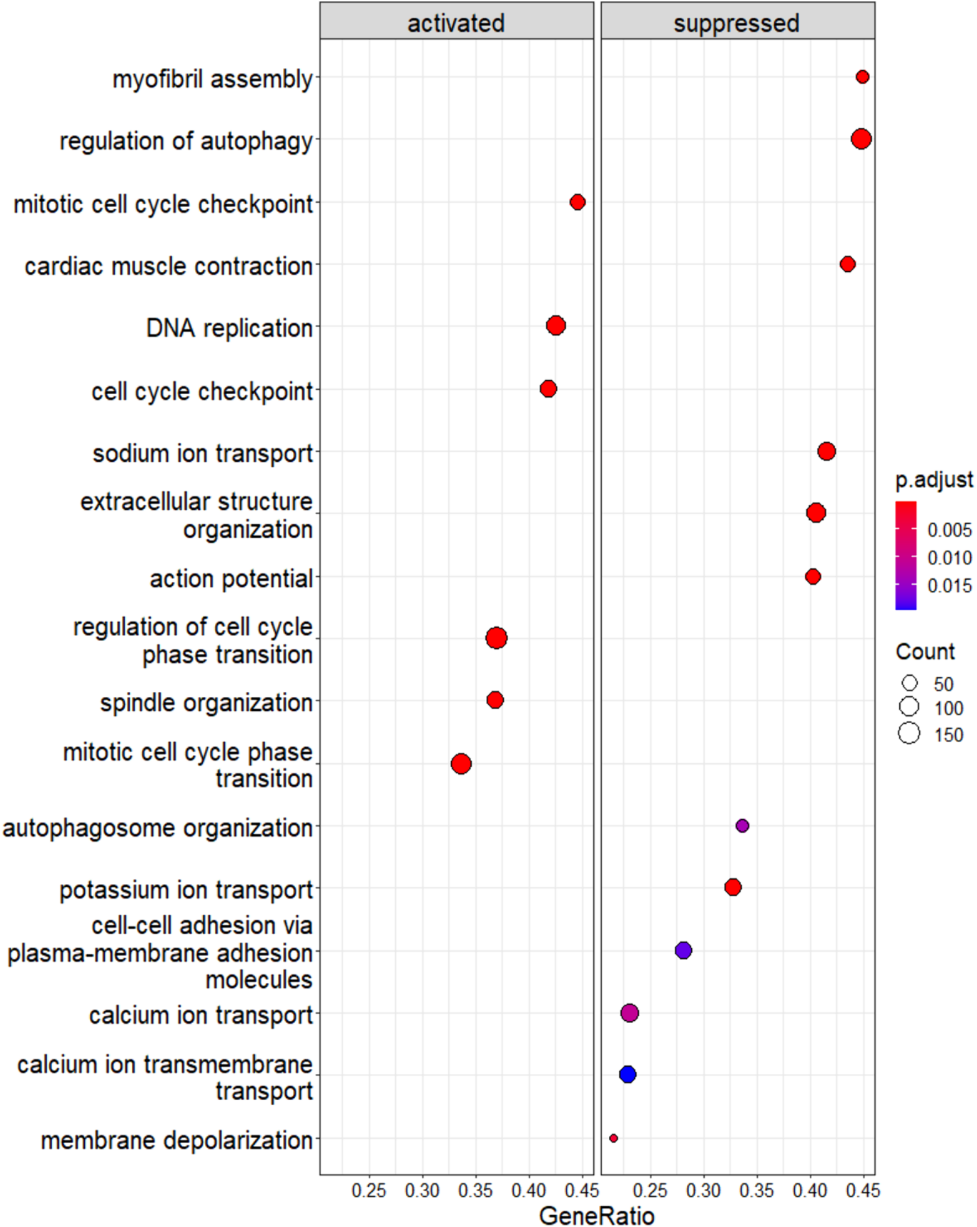
Results for gene set enrichment analysis (GSEA). Significant activated and suppressed GO terms were considered if *p*‐adjust values were ≤0.05. The dot size represents the number of genes associated with the GO term, the dot color represents adjusted *p*‐values.

To evaluate the biologic pathways altered by R1267Q mutation we performed Gene set (pathway) enrichment analyses (GSEA) using Gene Ontology database (Biological Process, Cellular Component, and Molecular Function). As expected, among suppressed pathways we identified several already known features of the *FLNC*‐associated cardiomyocyte disfunction, such as myofibril assembly, cardiac contraction, and extracellular matrix organization. In addition, among the suppressed pathways there were: action potential and the transport of sodium, potassium, and calcium ions, which well agrees with the data obtained by electrophysiological studies (Figure 4). On the other hand, R1267Q *FLNC* variant was found to be associated with cell cycle activation, DNA replication, and mitotic process in vitro.

### 3.3 Characterization of V2264M *FLNC* variant

#### 3.3.1 Calcium imaging

**FIGURE 5:**
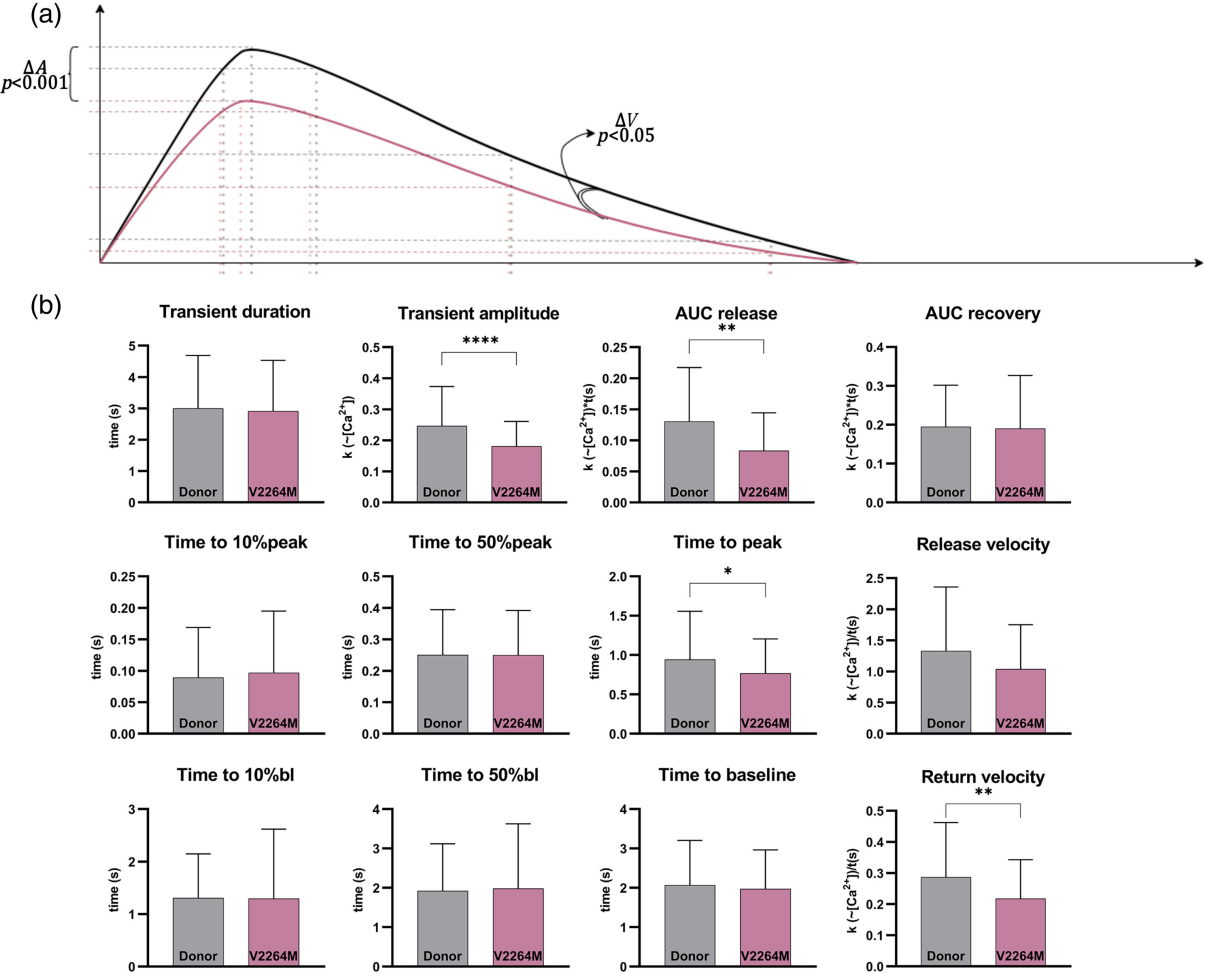
Calcium transients of V2264M hiPSC‐CMC and Donor hiPSC‐CMC. Black plot—health donor hiPSC‐CMC (*n* = 129); red plot—V2264M hiPSC‐CMC (*n* = 65). (a) Dynamics of calcium transients; dotted lines—time points of calcium transient (10%, 50%, 90%, and peak); *A*—calcium transient amplitude; *V*—velocity; different properties are specified; *p*‐value marked under each parameter. (b) Parameters of calcium transient. AUC—area under curve. *p*‐value: * −<0.05; ** −<0.01; *** −<0.001; **** −<0.0001. The columns are presented as the mean and the bars correspond to SD.

Calcium transients of V2264M hiPSC‐CMC were more similar to Donor hiPSC‐CMC compared to R1267Q hiPSC‐CMC (Figure 5a). In V2264M hiPSC‐CMC only transient amplitude was found to be significantly decreased, resulting in significant decrease of AUC release (Figure 5b). We also detected significant decrease in return velocity V2264M hiPSC‐CMC which may reflect decreased transient amplitude under the same time to baseline and, generally, transient duration in comparison with Donor hiPSC‐CMC (Figure 5b).

We also detected the decrease in protein amount of STIM1 and TRPC1 using Western blot (Supplemental Figure 2), which further supports the alterations in calcium homeostasis in V2264M hiPSC‐CMC on functional and protein levels.

#### 3.3.2 Electrophysiology

**FIGURE 6:**
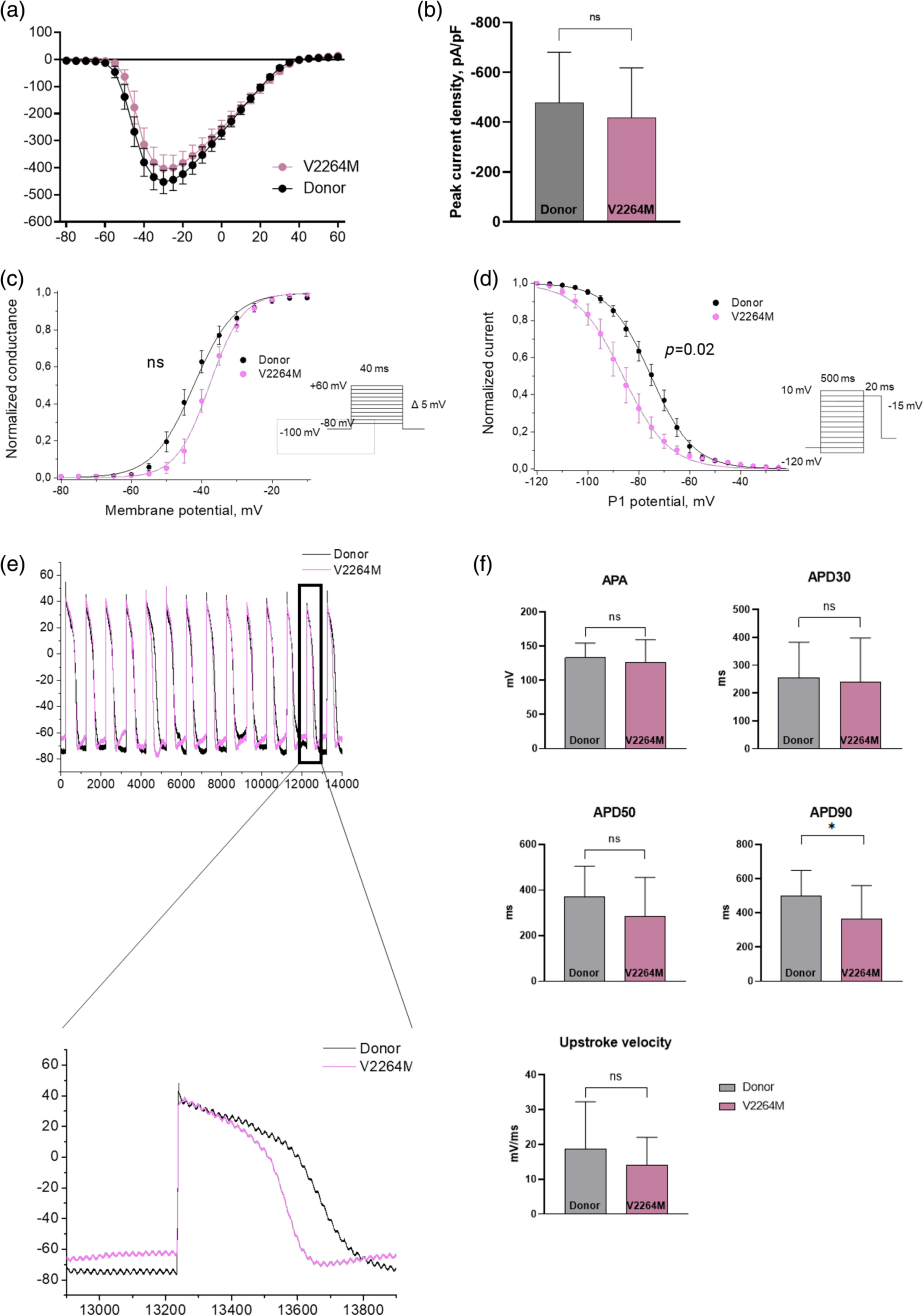
Biophysical characteristics of sodium current and action potential in V2264M hiPSC‐CMC. Black plot—health donor hiPSC‐CMC (*n* = 23); red plot—V2264M hiPSC‐CMC (*n* = 14). (a) Volt–Ampere characteristics of sodium current. (b) Peak of sodium current density. (c) Steady‐state activation of sodium current. (d) Steady‐state inactivation of sodium current. (e) Representative traces of action potentials. (f) Parameters of action potential. *p*‐value: *−0.05; **−<0.01; ***−<0.001; ****−<0.0001. The columns are presented as the mean and the bars correspond to SD.

In V2264M hiPSC‐CMC peak sodium current density was not significantly changed compared to Donor hiPSC‐CMC (Figure 6a, b). Similarly, voltage‐dependency of steady‐state activation does not show any alterations in V2264M hiPSC‐CMC compared to Donor CMC (Figure 6c). Voltage‐dependency of steady‐state inactivation of V2264M hiPSC‐CMC demonstrated significant 11 mV hyperpolarizing shift (Figure 6d). In addition, we detected a significant decrease of action potential duration (APD90) in V2264M hiPSC‐CMC, possibly, due to enhanced inactivation of sodium channels (Figure 6e, f).

#### 3.3.3 Pathway analysis of transcriptional changes in V2264M hiPSC‐CMC

To capture biological differences between V2264M hiPSC‐CMC and Donor hiPSC‐CMC, we analyzed functional pathways within DEGs. The GO annotations of DEGs included three parts‐ Biological process, cellular component, and molecular function, which were used to analyze the functional enrichment of DEGs.

**FIGURE 7:**
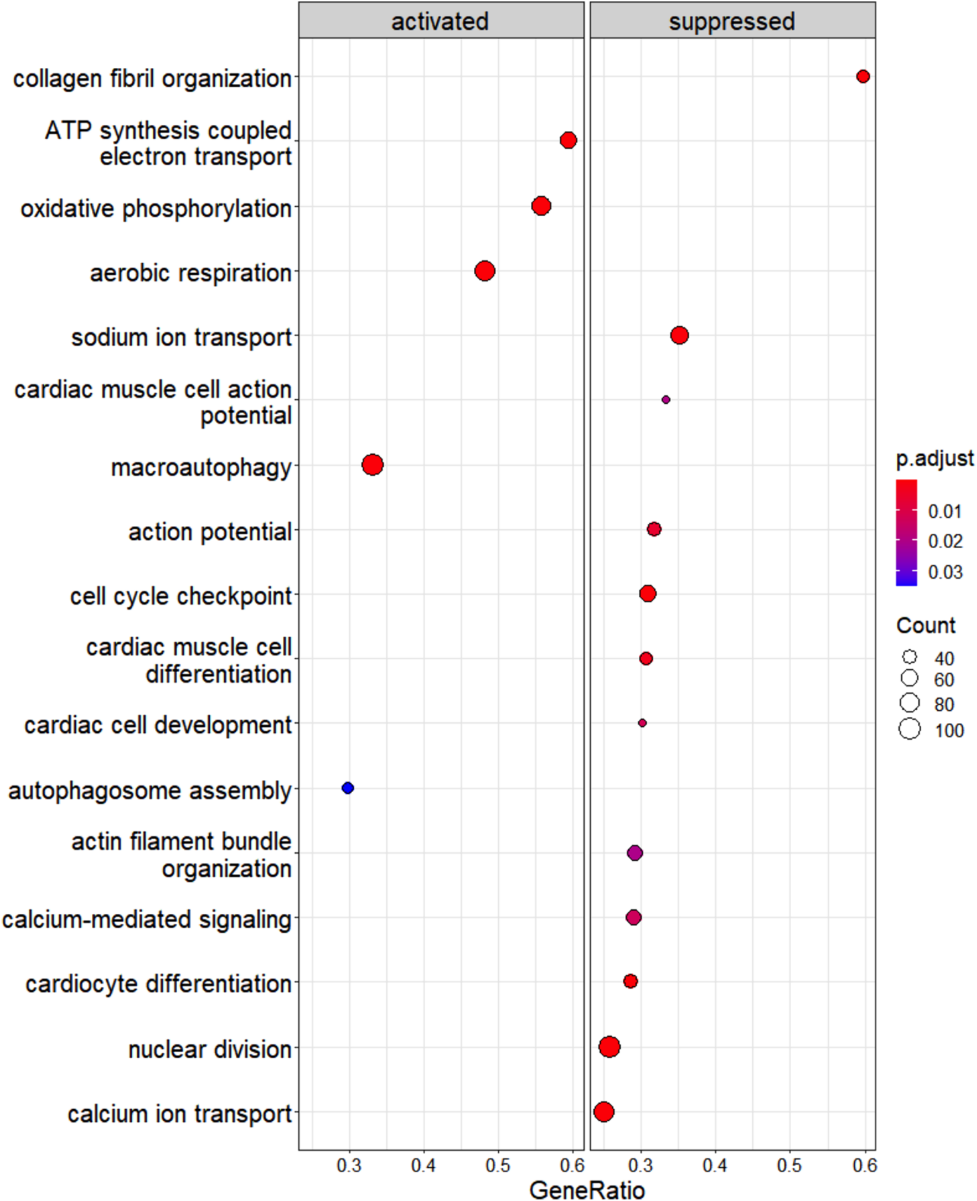
Results for gene set enrichment analysis (GSEA) for differentially expressed genes. Pathway analysis was performed on downregulated and upregulated DEGs in V2264M/Donor comparison.

In opposite to R1267Q hiPSC‐CMC, in V2264M hiPSC‐CMC DEGs involved in cell cycle were downregulated, as well as pathways linked to cell adhesion, cardiac cell differentiation and contraction, potassium and sodium ion transport and action potential (Figure 7). Among upregulated pathways we detected those linked to protein synthesis, translation and regeneration.

**FIGURE 8:**
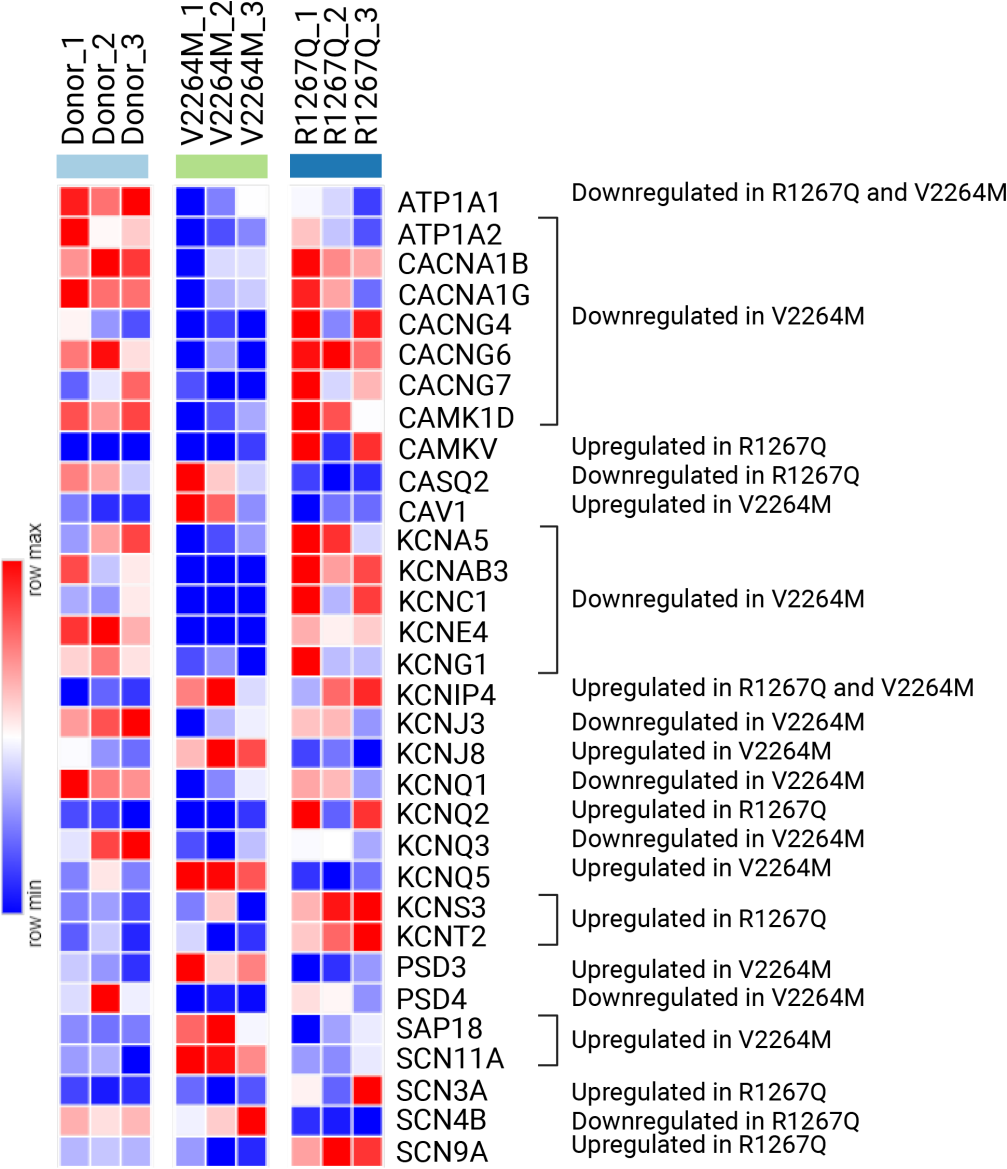
Heatmap comparison of DEGs related to ion channel function, action potential, and calcium homeostasis for V2264M hiPSC‐CMC, R1267Q hiPSC‐CMC, and Donor hiPSC‐CMC.

Overall, V2264M hiPSC‐CMC demonstrated higher number of downregulated DEGs compared to R1267Q hiPSC‐CMC, some of them being common for both patient‐specific cell lines (Figure 8). Of note, two patient‐specific cell lines demonstrated mainly line‐specific alterations in ion channel gene expression, except for several genes such as *ATP1A1* and *KCNIP4*.

To sum up, we detected changes in action potential, Nav1.5 function, and calcium transients in patient‐specific cardiomyocytes carrying R1267Q and V2264M *FLNC* variants. Along with several common alterations such as action potential shortening and alterations in several gene expressions, these two hiPSC‐CMC lines demonstrate distinct sets of functional and expression alterations, possibly, leading to different disease molecular mechanisms and clinical phenotypes.

### 3.4 Myofibrillar organization and structure in R1267Q hiPSC‐CMC and V2264M hiPSC‐CMC

**FIGURE 9:**
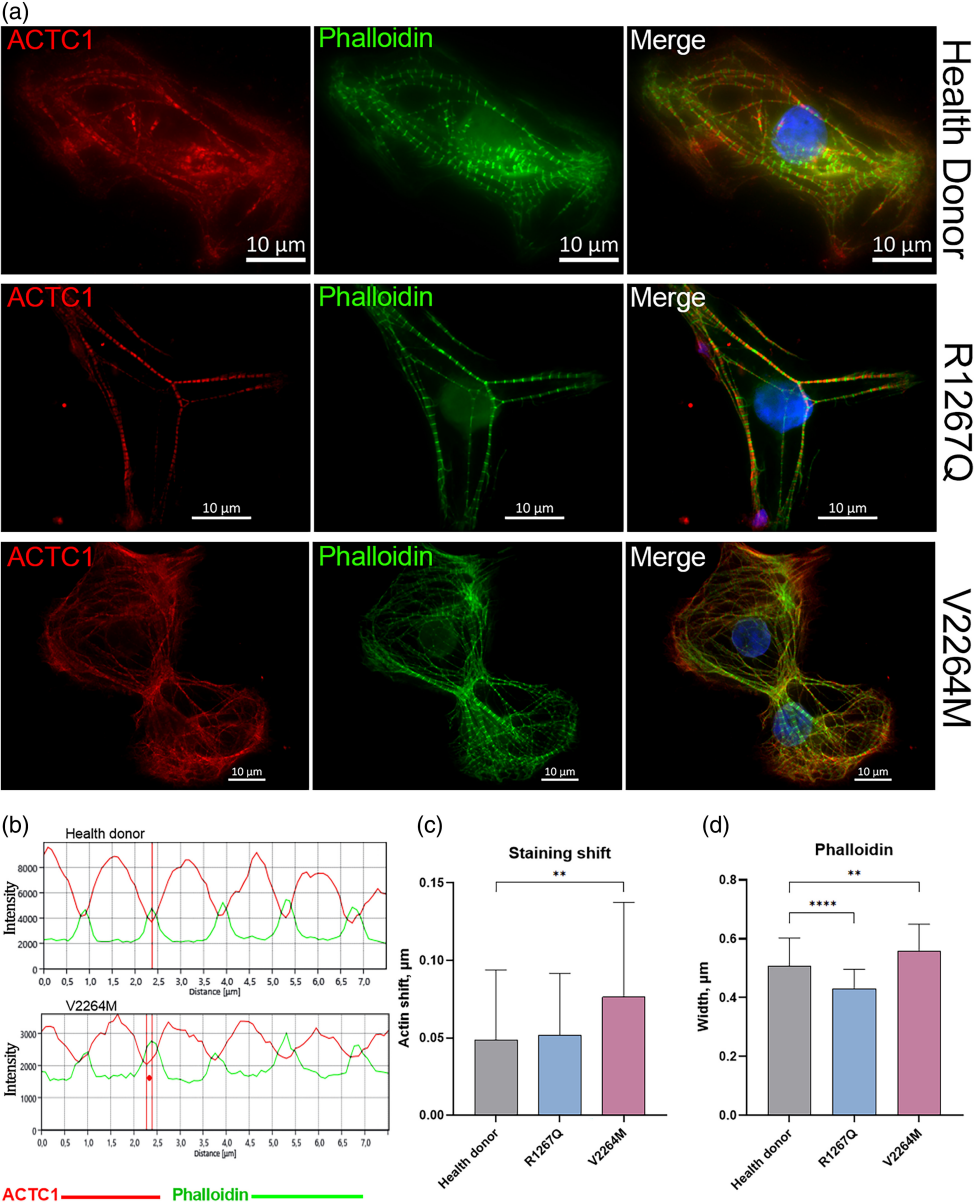
(a) Representative immunofluorescence staining of cardiac alpha‐Actin (ACTC1—Red) and phalloidin staining (Phalloidin—Green). The top row—Hipsc‐CMC cells of Health Donor; The middle row—Hipsc‐CMC cells from patient with R1267Q mutation. The bottom row—Hipsc‐CMC cells from patients with V2264M mutation. (b) Representative sample of the intensity profile (red graph—Cardiac alpha‐Actin; green graph—Phalloidin) in donor cells and in hiPSC‐CMC cells from patients with V2264M mutation. Caliper X between channels' extremums reflects actin shift in absolute value. (c) Measured actin shifts in donor and patient hiPSC‐CMC cells. (D) Width of phalloidin hyperfluorescent stained (*n* = 71; 103; 59 respectively). Images were acquired using consistent shooting parameters. The columns are presented as the mean and the bars correspond to SD.**p‐value <0.01, ****p‐value <0.0001.

Actin filament organization was analyzed using double‐staining with phalloidin and anti‐alpha‐actin antibodies in control and patient hiPSC‐CMC. Phalloidin staining rendered clear Z‐disk hyperfluorescent due to intensive actin cross‐linkage (Figure 9a). In contrast, anti‐cardiac alpha‐actin antibodies did not interact with Z‐disk area to the same extend, possibly, due to high protein density of this area and poor epitope availability. Accordingly, minimum anti‐cardiac alpha‐actin immunofluorescence intensity is detected at the sites of maximum phalloidin staining, which allows to measure the shift between nearest extremums (Figure 9b). We observed increased of actin shift in V2264M hiPSC‐CMC compared to donor cells. At the same time there was not significant difference between R1267Q hiPSC‐CMC and control cells (Figure 9c). At the next step we analyzed the width of hyperfluorescent phalloidin‐positive zone corresponding to Z‐disk and detected its expansion in V2264M cardiomyocytes compared to control cells and, in opposite, its reduction in R1267Q cardiomyocytes (Figure 9d).

**FIGURE 10:**
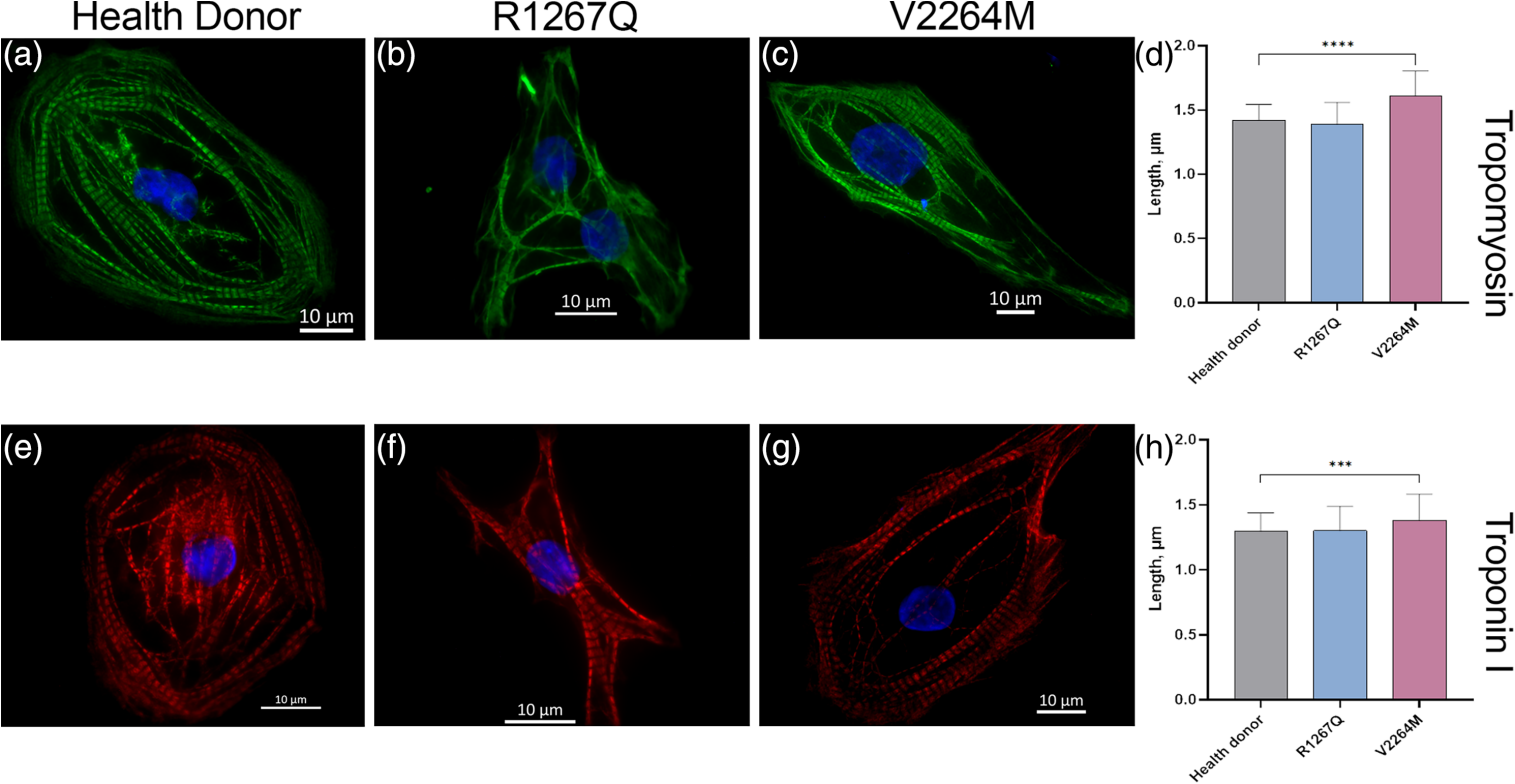
Length of TNNI3 and TPM1 fluorescent stained zone. (a)–(c) Representative immunofluorescent staining of tropomyosin with fluorescent intensity profile in control cells, FLNC‐R1267Q and ‐V2264M hiPSC‐CMC respectively; (e)–(g) representative immunofluorescent staining of tropomyosin with fluorescent intensity profile in control cells, FLNC‐R1267Q and ‐V2264M hiPSC‐CMC respectively; (d), (h) length of tropomyosin (*n* = 122; 89; 109, respectively) and troponin I (*n* = 54; 74; 59, respectively) peaks. The columns are presented as the mean and the bars correspond to SD. ***p‐value <0.001; ****p‐value <0.0001.

Length of troponin I and tropomyosin staining were measured using profile intensity instrument. We did not detect any visual structural differences between control donor cells and patient cells (Figure 10a–c and e–g). However, the analysis of intensity profiles revealed an expansion of the staining zone of both, troponin I and tropomyosin in V2264M hiPSC‐CMC (Figure 10d, h).

**FIGURE 11:**
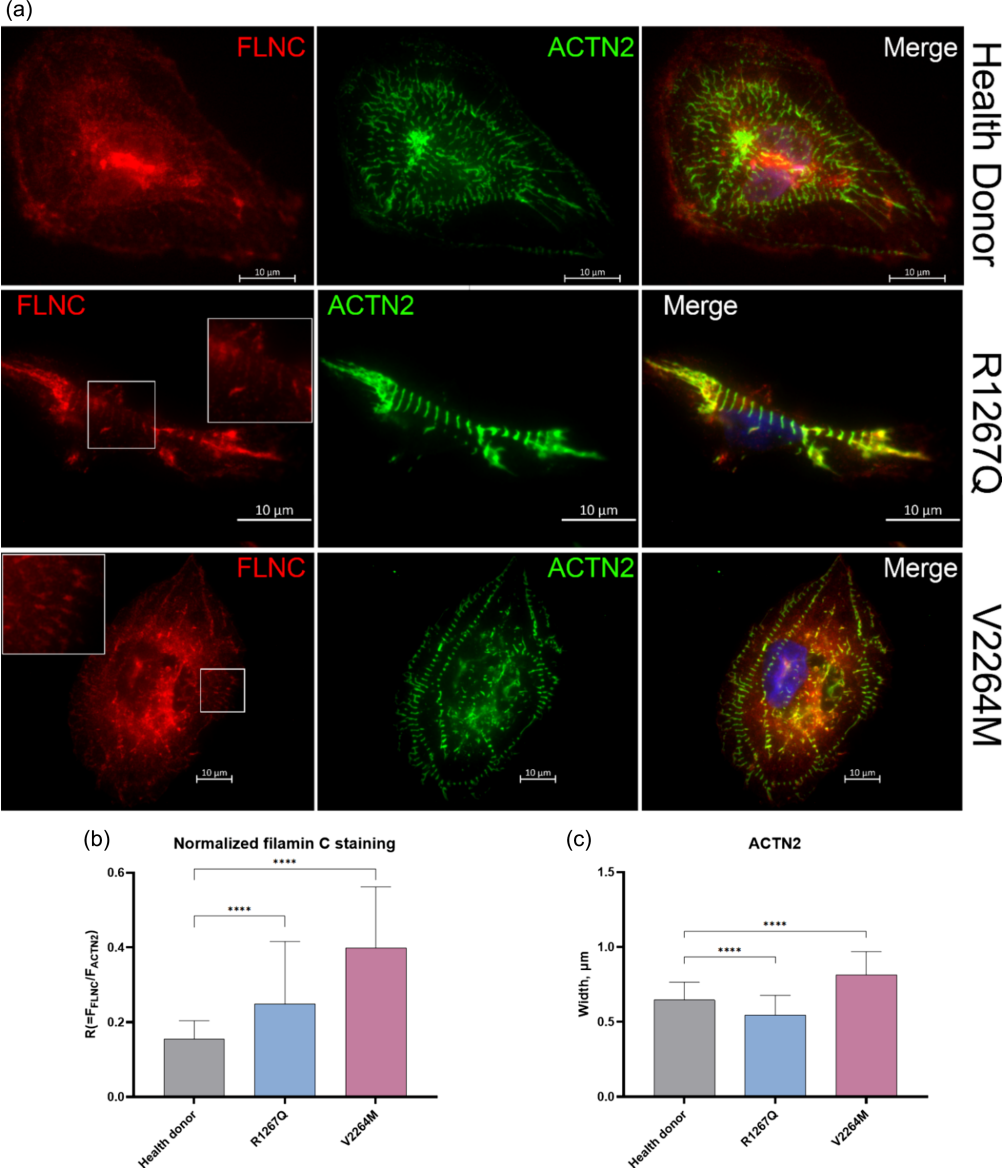
(a) Representative immunofluorescence staining of Filamin C (FLNC—red) and sarcomeric α‐actinin (ACTN2—green). The top row—hiPSC‐CMC cells of Health Donor; the middle row—hiPSC‐CMC cells of patient with R1267Q mutation; the bottom row—hiPSC‐CMC cells of patient with V2264M mutation, white square regions highlight FLNC striates co‐localized with ACTN2. The areas of filamin C visualization are highlighted by white rectangles. (b) Intensity of filamin C fluorescence staining normalized to α‐actinin staining. (c) Width of α‐actinin fluorescent stained (*n* = 82; 75; 93 respectively). Images were acquired using consistent shooting parameters. The columns are presented as the mean and the bars correspond to SD. ****p‐value <0.0001.

Filamin staining using immunocytochemistry with anti‐Filamin C antibody is not widely used due to the poor epitope availability. For this reason, most Filamin C visualization on cellular level is performed using tag epitopes, for example, GFP (Leber et al., 2016). In our study, we applied an antigen retrieval strategy using urea buffer in attempt to achieve specific Filamin C staining. We were able to visualize weak cross‐sectional Filamin C pattern in patient's hiPSC‐CMC in colocalization with α‐actinin, but not in the control donor hiPSC‐CMC (Figure 11a). The intensity of Filamin C fluorescence was assessed by normalizing to the intensity of α‐actinin fluorescence (assuming it to be the same in all samples as imaging was performed under the same conditions). We detected the increase in fluorescence intensity of Filamin C both in R1267Q hiPSC‐CMC and V2264M hiPSC‐CMC compared to control cells (Figure 11b). In addition, Z‐disk area was measured by ACTN2 staining leading to similar results received by phalloidin staining: Z‐disk area was expanded in V2264M hiPSC‐CMC compared to healthy donor cells, while R1267Q demonstrated the width reduction.

Thus, using immunofluorescent staining with anti‐myofilament antibodies we detected the decrease of Z‐disk area in R1267Q hiPSC‐CMC and increase in V2264M hiPSC‐CMC, supporting the hypothesis of distinct impact of R1267Q and V2264M mutations on cytoskeleton and sarcomere architecture. These data were further confirmed by increased degree of Filamin C epitope availability in mutant cells detected by anti‐Filamin C staining which can reflect the misfolding of mutant Filamin C which makes the epitope more accessible for the antibody.

## 4 DISCUSSION

*FLNC* pathogenic variants were widely recognized in connection to cardiac and skeletal muscle disorders. With regard to different structural and functional molecular effect of FLNC mutations, the final clinical phenotype can result either from protein aggregation and defects of proteostasis (restrictive cardiomyopathy or myofibrillar myopathy), or from filamin insufficiency due to loss of function effects of mutation. As a consequence, patient's prognosis and potential treatment options can differ substantially, leading to implementation of different risk scales and indications for device therapy (Setti et al., 2024).

The application of hiPSC‐CMC approach using patient‐specific cell lines allows to dissect these distinct mechanisms, especially in combination with functional electrophysiological and Ca‐oscillations studies. Since *FLNC*‐associated cardiomyopathies have a well‐proved increased risk of arrhythmic events, the combination of electrophysiological studies with expression analysis can uncover the bases for ion channel disfunction in patients with different types of *FLNC* mutations.

In our study we focused on two FLNC variants previously described by our group and associated either with arrhythmogenic (R1267Q) or restrictive (V2264M) cardiomyopathy (Kofeynikova et al., 2023; Muravyev et al., 2022; Song et al., 2007). The first variant—R1267Q—is the only one localizing in Ig11 repeat of ROD1 domain of filamin C. Several other mutations localizing in the neighboring domains have been described associated with all types of cardiomyopathies—dilated, arrhythmogenic, hypertrophic, and restrictive (Eden & Frey, 2021), therefore the detailed molecular mechanisms of R1267Q pathological effect cannot be simply attributed to the variant location. The second variant—V2264M—localizes into Ig20 repeat of ROD2 domain which is known to interact with myopodin and myotillin and calsarcines (Eden & Frey, 2021). One can speculate that destabilization of these interactions important for entire Z‐line architecture can lead to Z‐disk derangements, damage, and protein aggregation, often observed in patients with *FLNC*‐associated RCM. Thus, the molecular mechanisms of deleterious effect of these two *FLNC* variants can be quite different. Using hiPSC‐CMC approach we demonstrated that R1267Q variant cause more severe electrophysiological dysfunction, leading to prolongation of calcium transient time, impairment of sodium current (Nav1.5) density of alterations of sodium channel activation. These functional changes were supported by changes in transcriptome profile involving downregulation of genes linked to action potential and sodium transport as well as structural cardiomyocyte genes. For example, we detected dysregulation in expression of *SCN4B*, *SCN3A*, and *SCN9A* in R1267Q hiPSC‐CMC and upregulation of several potassium channels such as *KCNS3* and *KCNT2*. Even though these genes are normally expressed in cardiomyocytes at very low levels, their upregulation can result from alterations in normal CMC ion channel traffic, positioning, and function due to *FLNC* defects. Similar changes were observed for Ca‐homeostasis‐related genes. For example, downregulation of *CASQ2* and *HRC* detected in R1267Q hiPSC‐CMC, but not in V2264M hiPSC‐CMC can be linked to prolongation of all duration parameters of calcium transients (Song et al., 2007).

In contrast to R1267Q hiPSC‐CMC, V2264M hiPSC‐CMC demonstrated less functional alteration in ion channel functions and calcium oscillations. This corresponds to the mail clinical phenotype of V2264M, which is restrictive cardiomyopathy associated with protein aggregate formation (Kiselev et al., 2018; Onnee et al., 2024; Sellung et al., 2023). Accordingly, transcriptomic analysis of V2264M variant uncovered pathways, associated with protein synthesis and ribosomal function. Of note, the most significant transcriptional changes, linked to V2264M and associated with ion channels involved downregulation of various types of potassium channels (Figure 8) along with decreased amount of calcium‐homeostasis proteins demonstrated by Western blot. Taking into account minimal functional alterations of Na_v_1.5 and calcium homeostasis in V2264M hiPSC‐CMC, we speculate that these changes called for compensation of deleterious molecular effects caused by *FLNC* mutation in regard to ion channel dysfunction.

In spite of different electrophysiological properties and transcriptional changes caused by two different FLNC mutations, we detected several genes related to ion‐homeostasis whose expression changes similarly in both patient‐specific cell lines. Thus, downregulation of *ATP1A1* and *ATP1A2* encoded for Na^+^/K^+^ATPase was detected in both cases, as well as upregulation of *KCNIP4*. These expressional changes can modulate the function of outer membrane ion channels making further impact on electrophysiological consequences of *FLNC* variants.

Of note, we were not able to detect any FLNC‐containing protein aggregates neither in R1267Q—nor in V2264M hiPSC‐CMC despite the fact that protein aggregate formation is one of the leading molecular mechanisms of filaminopathies, espacially in case of RCM and HCM (Kiselev et al., 2018; Onnee et al., 2024; Sellung et al., 2023). The possible explanation for this fact can be the increased in autophagy flux in mutant CMC compensating the protein aggregation on the initial steps. This mechanism was earlier demonstrated for desmin mutations and potentially can contribute for proteostasis compensation in FLNC‐mutant cells (Sukhareva et al., 2023). However, Filamin C misfolding is a typical consequence of several *FLNC* mutations (Verdonschot et al., 2020). Taking into account the obtained data on immunostaining using anti‐FLNC antibody, we propose that protein misfolding makes it less polymerized and more accessible for antibody binding and detection by immunofluorescent staining particularly at Z‐disk area, leading to the weak but positive staining in mutant cells and absence of staining in control CMC.

Cardiomyocyte maturation and development of myofilament contractile machinery are accompanied by formation of Z‐disks with their transition from irregular, impermanent, and granular structure toward well‐organized sarcomeric cross‐striation (Ahn et al., 1993; Fischman, 1967). Also, expansion of Z‐disk was described in nebulin‐deficient and myopalladin‐deficient cells (Filomena et al., 2020; Tonino et al., 2010), that implies poison effect of Z‐line protein derangements on sarcomere architecture and function. In our study we observed Z‐disk widening and enlargement in V2264M hiPSC‐CMC cardiomyocytes which can be attributed to insufficient Z‐area maturation and disorganization. This notion was confirmed using antibodies against sarcomeric tropomyosin and troponin I further supporting the hypothesis of myofilament disorganization due to V2264M mutation.

In summary, using patient‐specific induced pluripotent stem cell approach, functional studies, and transcriptomic analysis we suggested distinct molecular effects of two *FLNC* variants linked to different types of cardiomyopathies in terms of myofilament organization, electrophysiology, ion channel function, and intracellular calcium homeostasis providing the evidence for different molecular mechanisms of these disorders and uncovering the bases for their different clinical phenotypes.

## AUTHOR CONTRIBUTIONS

ESK, AKZ, MYS, and EGN contributed to the conception and design of the study, analysis, and interpretation of the data, and drafting of the manuscript. AAK, ENM, TMP, and ESV contributed to the study concept and research design and wrote the manuscript. KSS, EGN, KIP, and NLR took part in the analysis and interpretation of the data and have been involved in revising the manuscript critically. AAK, TLV, ASM conducted the experiments and performed the analysis and interpretation of the data. All authors have read and agreed to the published version of the manuscript.

## FUNDING INFORMATION

The present work was supported by Russian Science Foundation grant number 20–15‐00271П.

## CONFLICT OF INTEREST STATEMENT

The authors declare that the research was conducted in the absence of any commercial or financial relationships that could be construed as a potential conflict of interest.

## Supporting information


**Data S1:** Supporting Information

**Supplemental Table 1** List of Differentially expressed genes (upgerulated and downregulated).

## Data Availability

The data that support the findings of this study are available on request from the corresponding author. The data are not publicly available due to privacy or ethical restrictions.
