# Supplementary material for: Distinct molecular features of FLNC mutations, associated with different clinical phenotypes
Source: Cytoskeleton (Hoboken). 2024 Sep 24;82(3):158–74. doi: 10.1002/cm.21922 (PMC11904857; doi:10.1002/cm.21922)
Supplement: Supplementary file 1 — Data S1: Supporting Information [file CM-82-158-s002.docx]

Supplement


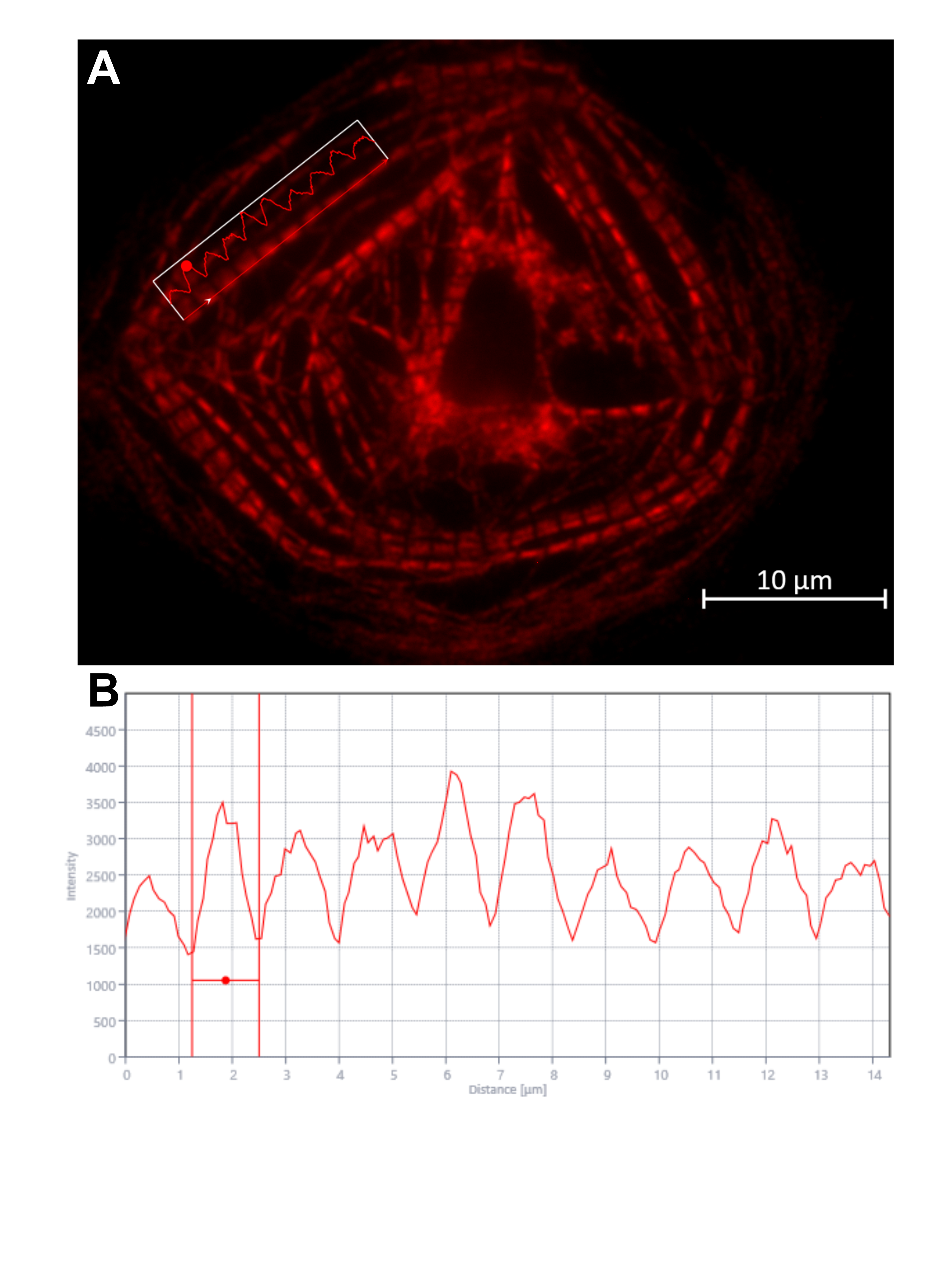


Supplemental Figure 1. Intensity profile measurement. **A)** Representative immunofluorescent staining of TNNI3 with fluorescent intensity profile in control CM-hiPSC; **B)** – An example of fluorescent intensity profile, caliper X of fluorescent intensity for obtained peaks.


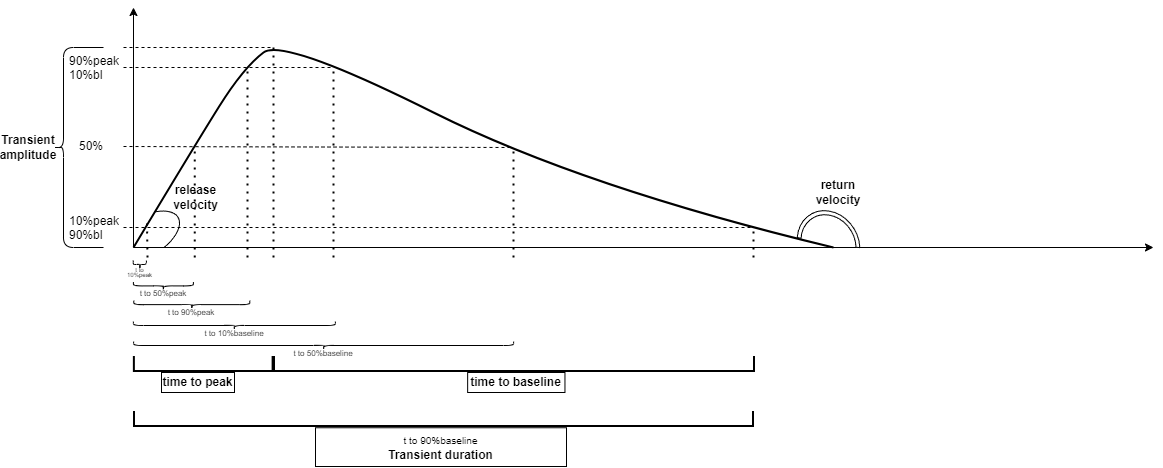


Supplemental Figure 2. Analysed parameters of calcium transient in health donor and FLNC-mutant hiPSC-CMC.


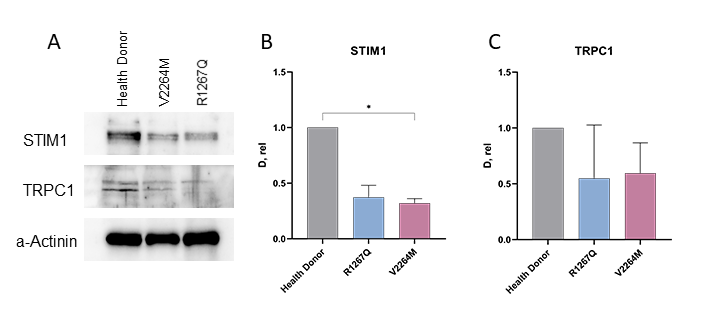


Supplemental Figure 3. **A)** Western blot images obtained using anti-STIM1 and anti-TRPC1 antibody. **B,C)** Decreased abound of STIM1 and TRPC1 proteins compared to control donor iPSC-CM. The normalization is performed compared to alpha-Actinin. The columns are presented as the mean and the bars correspond to SD.


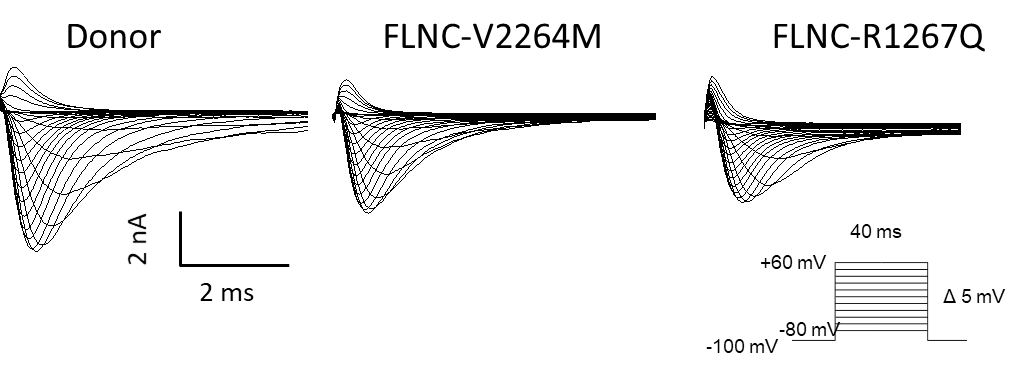


Supplemental Figure 4. Typical sodium current of health donor and FLNC-mutant hiPSC-CMC.

Supplemental material (Cardiogenic differentiation of iPSC)

In brief, hiPSCs were seeded in density 6x10^5 cell/3.8 cm^2 two days before induction of cardiogenic differentiation. 90-100% confluent cells were induced towards cardiogenic direction by changing growth medium (Essential 8) to differentiation medium (RPMI/B27ins-: Glutamax supplemented RPMI1640, B27 supplement without insulin, 100 U/ml penicillin, 100 μg/ml streptomycin (Thermo Fisher Scientific)) with 6 μM CHIR99021 (Selleckchem) (0 day). After 48 h previous medium was switched to RPMI/B27ins- medium containing 5 μM IWR1 (Stem Cell Technologies) (2 day). For the next 48 h, cells were cultured in RPMI/B27ins- medium. From day 6 to days 21-23 cells differentiated in RPMI/B27 medium (Glutamax supplemented RPMI1640, B27 supplement, 100 U/ml penicillin, 100 μg/ml streptomycin (Thermo Fisher Scientific)). Fresh RPMI/B27 medium was applied every two days.
